# Performance evaluation of a novel brain-dedicated SPECT system

**DOI:** 10.1186/s40658-018-0203-1

**Published:** 2018-03-01

**Authors:** M. K. Stam, E. E. Verwer, J. Booij, S. M. Adriaanse, C. M. de Bruin, T. C. de Wit

**Affiliations:** Department of Radiology and Nuclear Medicine, Academic Medical Center, PO box 22660, 1100 DD Amsterdam, The Netherlands

## Abstract

**Background:**

Single-photon emission computed tomography (SPECT) imaging is an important diagnostic tool for the early detection of the loss of nigrostriatal dopaminergic neurons in Parkinson’s disease (PD) and similar neurodegenerative disorders. Visualization and quantification of dopamine transporter (DAT) binding in the striatum is an established diagnostic tool to detect nigrostriatal dopaminergic degeneration. Given the small size of the striatum, high-resolution imaging is recommended. The InSPira HD system, a novel brain-dedicated SPECT scanner, allows for such detailed information with a spatial resolution down to ~ 3 mm full width at half maximum (FWHM). The current study examines performance of the InSPira HD for DAT imaging, by combining phantom tests from NU 1-2012 and NU 2-2012, and striatal scans.

Due to the unique geometry of the InSPira, and fixed acquisition and reconstruction settings, standard National Electrical Manufacturers Association (NEMA) testing is not applicable. Therefore, a combination of NU 1-2012 and NU 2-2012 standards were applied, with modifications to accommodate the InSPira HD. A small Jaszczak phantom with hot spheres and cold rod inserts was used to determine recovery coefficients, contrast, and uniformity. Spatial resolution was evaluated across the field of view (FOV) for point and line sources in air and water. A striatal phantom was used to model DAT imaging. A clinical, a high-resolution, and an experimental research reconstruction method were compared.

**Results:**

Acquired SPECT images demonstrated spatial resolution in air of ~ 3 mm in the center in the FOV for the high-resolution reconstruction approach. Spatial resolution in air for the clinical and research reconstruction approach was ~ 6–8 mm in the center of the FOV, which decreased in the transaxial plane with increasing radial distance from the center of the FOV. Reconstructed images of the uniform area of the Jaszczak phantom showed limited variability with a coefficient of variation of 2.6% for the clinical reconstruction and 3.0% for the research reconstruction. The ≥ 6-mm rod group and all spheres were resolved for the clinical and research reconstruction approaches. Recovery coefficients (RCs) for the Jaszczak phantom ranged from 0.49 to 0.89 (sphere diameters between 9.8 and 31.2 mm). RCs for the striatal phantom ranged from 0.50 to 0.55, with linearity of striatal ratios for a range of background concentrations (*R* = 0.97).

**Conclusions:**

Results from the phantom data demonstrated acceptable image quality for the InSPira HD system for DAT SPECT imaging in humans.

## Background

Single-photon emission computed tomography (SPECT) imaging is an important diagnostic tool for the early detection of the loss of nigrostriatal dopaminergic neurons in Parkinson’s disease (PD) and similar neurodegenerative disorders. The success of the dopamine transporter (DAT) targeted SPECT tracer ^123^I-ioflupane (^123^I-FP-CIT) for this purpose has been well established [1, 2]. Given the small size of the striatum, high-resolution imaging is recommended in diagnostic imaging of neurodegenerative disorders like PD and dementia with Lewy bodies (DLB) [2]. Because the spatial resolution that conventional SPECT cameras can achieve is limited (7–12 mm), dedicated high-resolution human brain SPECT imaging systems have been developed, e.g., the Neurofocus system (software upgrade of the Strichman Medical Equipment 810X, Medfield, Massachusetts, USA), which was installed in our institution back in 1990 [3].

**Fig. 1:**
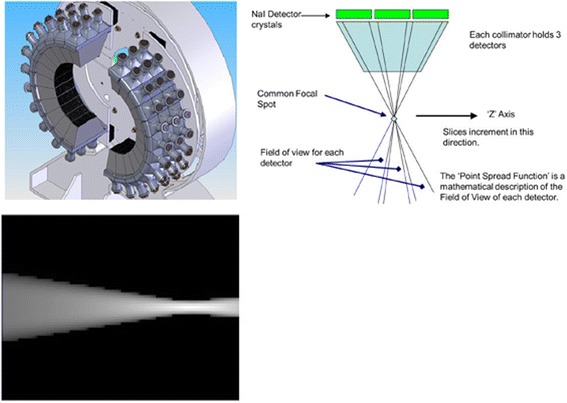
Schematic representation of the InSPira HD system showing the gantry without covers (left) and the collimator configuration (right). The collimator consists of three different detectors along the *z* direction. The point spread function (PSF) is given by the fractional solid angle of the rays that successfully pass through the collimator. Typically, each PSF has a focal spot where the value is at its maximum (below: 2D view of one of the InSpira PSF functions) (images obtained from Neurologica, USA)

The current study evaluates the performance and clinical applicability of a novel dedicated high-resolution brain SPECT imaging system, the InSPira HD (Neurologica, Boston, USA), with an aimed spatial resolution down to ~ 3 mm full width at half maximum (FWHM). High-resolution imaging is achieved by the unique design of the detector ring of the InSPira HD (Fig. 1). Such detailed information in DAT is especially important for the differential diagnosis of striatal dopaminergic loss in PD, progressive supranuclear palsy (PSP), and multiple-system atrophy (MSA) [4]. Different subregional losses of striatal DAT are observed in patients with PSP and MSA, compared to PD. In addition to detailed spatial information, quantification of striatal uptake is beneficial for differential diagnosis [4], and reduces equivocal reports [5]. The aim of the present study was to examine the performance of the system in more detail with respect to clinical DAT imaging. By scanning a small Jaszczak phantom, Sensakovic et al. [6] showed that the InSPira surpassed American College of Radiology (ACR) requirements. The present study aimed to reproduce previous findings by Sensakovic et al. [6] on visible spheres, sphere contrast, visible rod groups, and uniformity using a small Jaszczak phantom. Spatial resolution in air and water, as well as regional quantification was explored, focusing on DAT imaging by using a striatal phantom. Finally, the optimal reconstruction protocol was identified. Given the unique design of the InSPira HD, phantom tests from both National Electrical Manufacturers Association (NEMA) NU 1-2012 and NU 2-2012 were included, with necessary modifications, making direct comparisons to other SPECT systems difficult.

## Methods

The aim of the present study was to reproduce previous findings [6] and to evaluate additional phantom measurements relevant for DAT imaging. Therefore, in addition to typical NEMA testing setup for gamma cameras (NU 1-2012) [7], several tests were adapted from NU 2-2012 [8], originally designed to evaluate performance of PET systems, to augment conventional SPECT testing. It should be noted that extended modifications had to be made to NEMA testing, in order to accommodate for the geometry of the InSPira HD system. In addition to NEMA testing, striatal phantom measurements were performed and a DAT scan from a healthy control and a patient were retrospectively included to illustrate the clinical applicability of the InSPira HD system for DAT imaging. DAT imaging and processing were executed according to guidelines published by the European Association of Nuclear Medicine [9]. See below for details on the phantom measurements (Additional file 1).

### The InSPira HD system

High-resolution imaging is achieved by the unique design of the detector ring of the InSPira HD (Fig. 1). The detector ring consists of two clamshells, each containing 12 fanbeam collimators. At start position, the two clams touch and the collimators are focused at the center of the ring, achieving a focal point of 3 mm in diameter. During acquisition, the gantry rotates and simultaneously the two clams are moved outward leading the two focal points of the clam’s collimators to follow spiral trajectories over the field of view (FOV). The spatial resolution in the resulting image slice is therefore determined by the speed at which the clams move outward. After acquiring the first slice, the camera moves in axial direction to the position for acquiring the adjacent slice, and so on. A proprietary iterative reconstruction algorithm tailored to this unique method of spatial sampling is used to reconstruct the data into 3D images. This iterative reconstruction algorithm is based on a maximum a posteriori (MAP) estimation. It includes a point spread function (PSF), which is defined as the detector response to an impulse activity source point placed in the scanner FOV. Attenuation correction is performed using either a CT previously obtained from the patient/phantom or a deformable CT template. Scatter correction was not available on the system and therefore not performed.

### Acquisition and reconstruction parameters

For the striatal phantom and clinical DAT imaging, a slice time of 180 s and slice spacing of 4 mm was used, resulting in a ~ 30 min scan duration, while maintaining sufficient spatial resolution. For all other scans, a slice time of 240 s and a slice spacing of 3.125 mm were used. Energy windows were 20% centered around 140 and 159 keV for the ^99m^Tc and ^123^I energy windows. Three reconstruction approaches were investigated. A “clinical” reconstruction approach, with 60 iterations and voxel size of 2.083 × 2.083 x slice thickness mm^3^, as recommended by the vendor. A “research” reconstruction using a developers program (80 iterations with an increased sampling rate in projection space; voxel size 3.125 × 3.125 × slice thickness mm^3^). To assess maximum achievable resolution of the system a “high-resolution” (1000 iterations) reconstruction approach was examined, which did not include attenuation correction, which was only used for point and line sources (voxel size of 2.083 × 2.083 × slice thickness mm^3^). For all other measurements, attenuation correction was used in the reconstruction by importing a CT-scan of the phantom.

### Phantom preparation and positioning

#### 3D resolution in air

The tips of three capillaries with an inner diameter of 0.8 mm were filled with ^99m^Tc (~ 1.5 MBq) to create point sources to assess resolution in air in *x*/*y* in-plane and *z* axial direction. The sources were positioned in the same coronal plane such that the middle source was positioned in the center of the FOV, one source was positioned approximately 7.3 cm left and 5.0 cm cranial of the central source, and one source was positioned 7.3 cm right and 5.0 cm caudal. All capillaries were attached to the table such that the point source was surrounded by air. A scan of 40 slices was acquired. Resolution was determined by calculating FWHM in three dimensions.

#### Resolution in air

Spatial resolution in air across the FOV was calculated using line sources in air. Ten capillaries with an inner diameter of 0.2 mm were filled with ^99m^Tc (~ 1.2 GBq/ml). The capillaries were inserted into holes drilled into a round acrylic disk from where they suspended in air, located at the center of the FOV, 2, 4, 6, and 8 cm to the left and right, 3, 6, and 9 cm above and below the center. A single slice scan was recorded. FWHM in *x* and *y* direction was calculated.

#### Resolution in water

Three line sources in water were used to determine the spatial resolution in a scattering medium. Three capillaries with inner diameter of 0.8 mm were filled with ^99m^Tc (~ 23 MBq) and inserted into a cylindrical water-filled phantom with a diameter of 14 cm. One capillary was placed in the center of the cylinder and the two capillaries were placed at 5 cm distance from the center with a 90° angle between them. First, a scan with no background activity concentration in water was performed. Next, two scans with background activity concentration of 0.1 and 1.0% of total activity in the capillaries were performed. Average and standard deviation (SD) FWHM in *x* and *y* direction was calculated for three consecutive slices. For scans with background activity, mean background was defined in three circular ROIs with diameter of 12.5 mm in the slice with peak values, and subsequently subtracted from all pixels, before calculating FWHM.

#### Contrast

A Jaszczak phantom with a diameter of 140 mm was custom made by Neurologica to fit the size of the InSPira HD. Either fillable spheres (31.2, 24.9, 19.7, 15.7, 12.4, and 9.8 mm inner diameter) or rods (11, 9.5, 7, 6, 4.5, and 3 mm diameter) were inserted. Spheres were filled with a 37.5 kBq/ml ^99m^Tc solution and the background compartment with a 8.4 kBq/ml ^99m^Tc solution, resulting in a sphere-to-background ratio of 4.4 to 1. A scan of 30 axial slices centered at the hot spheres was acquired. On the reconstructed image, a series of circular regions of interest (ROIs) were projected onto the spheres, selecting the slice with peak value. Six ROIs were projected between the spheres to determine background concentration (all 17.6 mm diameter, except one with 13.6 mm diameter in order to fit between the two largest spheres). Contrast recovery coefficients (CRC) were then calculated using Eq. 1 below, where pixcount_sphere,J_ represents the measured activity concentration in sphere *J* and activity_sphere,J_ represents the actual activity concentration in the sphere *J*. Similarly, pixcount__background_ represents the mean activity concentration from the six background ROIs, and activity__background_ is the actual activity concentration in the background.

1 $$ \mathrm{CRC}=\frac{\mathrm{pixcoun}{{\mathrm{t}}_{\mathrm{sphere}}}_J/\mathrm{activit}{{\mathrm{y}}_{\mathrm{sphere}}}_J}{\mathrm{pixcoun}{\mathrm{t}}_{\mathrm{background}}/\mathrm{activit}{\mathrm{y}}_{\mathrm{background}}} $$

#### Visual assessment of spatial resolution

The custom Jaszczak phantom with the cold rods insert was used to assess visual resolution. The phantom was filled with a solution of ^99m^Tc at a concentration of 46.76 kBq/ml. From the reconstructed image, four adjacent slices that showed the rods section with the best contrast were selected and summed. The resulting image was examined visually to identify the smallest rods that could be discerned.

#### Uniformity

The uniform area of the custom Jaszczak phantom was used for assessment of uniformity. Four slices containing the uniform area were summed. Uniformity was assessed qualitatively by plotting a 6.25 mm wide profile across the phantom. A quantitative measure of uniformity (coefficient of variation (CV)) was calculated using Eq. 2, where SD_N_ROI is the standard deviation of the voxel values in a 100-mm-diameter circular ROI, placed within the central slice and Mean_N_ROI the mean voxel value.

2 $$ \mathrm{CV}\ \left(\%\right)=\frac{{\mathrm{SD}}_{\mathrm{N}}\mathrm{ROI}}{{\mathrm{Mean}}_{\mathrm{N}}\mathrm{ROI}}\times 100\% $$

#### Striatal phantom

A striatal head phantom (anthropomorphic striatum phantom; Radiology Support Devices Inc., Long Beach, CA, USA [RS-901 T]) was filled with ^123^I dissolved in water at a concentration of 9.1 kBq/ml for the background, and 42 and 32 kBq/ml for the left and the right striatal compartment, respectively, yielding a striatal-to-background ratio of 4.6:1 and 3.5:1 [10, 11]. In addition, measurements with background concentrations of ~ 5 and ~ 15 kBq/mL were included to assess linearity of quantitative values. The Brain Registration and Analysis Software Suite (BRASS™, HERMES Medical Solutions, Sweden) was used, which fits the data to a template containing a number of volumes of interest (VOIs). CRC was calculated for left and right striatal VOIs using Eq. 1. The specific to non-specific binding ratio was calculated in bilateral striatum as follows: (total binding in striatum–non-specific binding in occipital cortex) /(non-specific binding in occipital cortex). Linearity of quantitative values (i.e. the association of the specific to non-specific binding ratio, with the ratio of the true activity concentration) was assessed using Pearson’s correlation.

#### Typical clinical example

To illustrate the clinical applicability of the InSPira HD system, we retrospectively included a DAT scan from a healthy control and a patient. The patient was scheduled for a routine ^123^I-ioflupane SPECT scan on the NeuroFocus system for clinical evaluation of possible parkinsonism, and the healthy control participated in a research study on the NeuroFocus. For both subjects, the scan on the InSPira was obtained after the NeuroFocus scan was completed (approx. 4 h after the injection of ~ 111 MBq ^123^I-ioflupane). Acquisition parameters for the InSPira HD were adopted from the standard clinical acquisition parameters on the NeuroFocus; a slice timing of 180 s and slice spacing of 4 mm was used for both systems resulting in a ~ 30-min scan duration, whilst maintaining sufficient spatial resolution. All procedures performed in studies involving human participants were in accordance with the ethical standards of the institutional committee.

## Results

For an overview of representative figures of all phantom SPECT images acquired, please see Additional file 2.

### 3D resolution in air

3D resolution varied slightly between the three sources, with best resolution for the center source. Resolution in *x*/*y* in plane, and *z* direction was comparable. As expected, best resolution was achieved using the high-resolution reconstruction, with FWHM of 2.4, 2.8, and 3.5 mm, in *x*, *y*, and *z* direction, respectively. Second best was the research software followed by the clinical software (Table 1).

**Table 1:** 3D resolution (FWHM) in air at three positions within the FOV for various reconstruction algorithms

	FWHM source 1 [mm]	FWHM source 2 [mm]	FWHM source 3 [mm]
Activity	1.13 MBq	1.77 MBq	1.95 MBq
Location	7.3 cm left and 5.0 cm caudal of source 2	Center FOV	7.3 cm right and 5.0 cm cranial of source 2
Clinical reconstruction algorithm	*X*: 6.9 *Y*: 7.4 *Z*: 6.6	*X*: 5.5 *Y*: 6.1 *Z*: 5.5	*X*: 7.8 *Y*: 8.5 *Z*: 7.8
High-resolution reconstruction algorithm	*X*: 3.3 *Y*: 3.9 *Z*: 3.9	*X*: 2.4 *Y*: 2.8 *Z*: 3.5	*X*: 4.0 *Y*: 4.7 *Z*: 5.9
Research reconstruction algorithm	*X*: 6.6 *Y*: 7.1 *Z*: 6.3	*X*: 5.2 *Y*: 5.7 *Z*: 5.1	*X*: 7.1 *Y*: 7.6 *Z*: 7.4

### Resolution in air

**Fig. 2:**
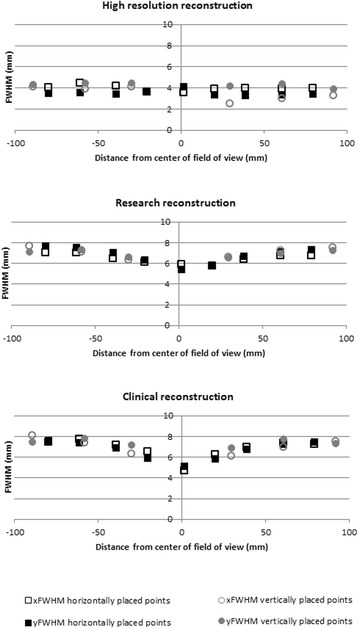
Spatial resolution (FWHM) in *x* and *y* direction as a function of radial distance to the center of the FOV, as measured with line sources in air. A total of nine sources were placed on the horizontal line, and six on the vertical line, with one source in the center of the field of view

Figure 2 shows the spatial resolution in air at various locations within the FOV. Consistent with the point source measurement, best results were obtained with the high-resolution clinical reconstruction followed by the research and the clinical reconstruction. An important observation is that FWHM appears to increase with increasing distance to the center of the FOV*.* The high-resolution reconstruction seemed not affected, with FWHM of 3.5 mm in *x*, and 4.1 mm in *y* direction in the center of the FOV, to 4.0 mm in *x* and 3.5 mm in *y* direction at the edge of the FOV. For the research reconstruction, resolution changed from 5.9 to 7.0 mm (*x* direction), and from 5.3 to 7.7 mm (*y* direction). For the clinical reconstruction, the effect was most pronounced, with changes in FWHM from 4.7 to 7.4 mm, and 5.1 to 7.6 mm, for *x* and *y* direction.

### Resolution in water

Resolution varied slightly between the three sources, with best resolution for the center source (6.18 mm for *x*, and 7.57 mm for *y* direction for the research reconstruction). Differences in FWHM between the three sources got more pronounced for the measurements including background activity concentration. For measurements with no background activity concentration in water, best results were found. This was followed by measurements with 0.1% background activity concentration. Measurements with 1.0% background activity concentration returned poorest FWHM. Best resolution was achieved using the research reconstruction followed by the clinical reconstruction (Table 2).

**Table 2:** Resolution in water (FWHM) in *x* and *y* direction for three line sources (~ 23 MBq) in water. Three measurements were executed; first with no activity concentration in water (no background [BG]), second with background activity concentration of 0.1% of activity in the line sources (0.1% BG), and third with background activity concentration of 1.0% of activity in the line sources (1.0% BG)

	FWHM line source 1 [mm]	FWHM line source 2 [mm]	FWHM line source 3 [mm]
Activity	22.3 MBq	22.6 MBq	24.4 MBq
Location	5 cm left from source 3	5 cm above source 3	Center FOV
Clinical reconstruction algorithm no BG	*X*: 8.11 ± 0.25 *Y*: 8.84 ± 0.28	*X*: 8.22 ± 0.03 *Y*: 7.62 ± 0.07	*X*: 6.18 ± 0.16 *Y*: 7.57 ± 0.19
Clinical reconstruction algorithm 0.1% BG	*X*: 9.28 ± 0.10 *Y*: 7.40 ± 0.09	*X*: 7.04 ± 0.04 *Y*: 9.21 ± 0.02	*X*: 6.21 ± 0.15 *Y*: 6.05 ± 0.09
Clinical reconstruction algorithm 1.0% BG	*X*: 9.34 ± 0.21 *Y*: 8.33 ± 0.50	*X*: 8.35 ± 0.55 *Y*: 10.60 ± 0.13	*X*: 9.48 ± 0.91 *Y*: 8.45 ± 0.76
Research reconstruction algorithm no BG	*X*: 7.53 ± 0.16 *Y*: 7.98 ± 0.22	*X*: 7.68 ± 0.05 *Y*: 6.94 ± 0.10	*X*: 5.74 ± 0.25 *Y*: 6.77 ± 0.10
Research reconstruction algorithm 0.1% BG	*X*: 8.95 ± 0.14 *Y*: 7.55 ± 0.11	*X*: 7.54 ± 0.02 *Y*: 9.16 ± 0.01	*X*: 6.79 ± 0.07 *Y*: 7.21 ± 0.08
Research reconstruction algorithm 1.0% BG	X: 8.07 ± 0.18 Y: 6.77 ± 0.45	X: 6.72 ± 0.37 Y: 8.27 ± 0.35	X: 7.54 ± 1.11 Y: 7.16 ± 0.67

### Contrast

**Fig. 3:**
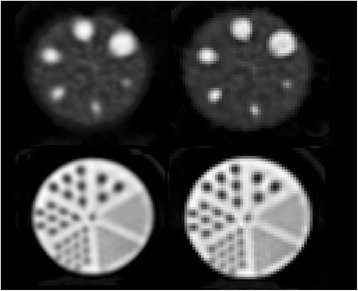
Reconstructed images of Jaszczak phantom, with the top plane showing the hot spheres, with a sphere-to-background ratio of 4.4 to 1, and the bottom plane showing the cold rods insert. Left column shows the clinical, and the right column shows the research reconstruction results

**Fig. 4:**
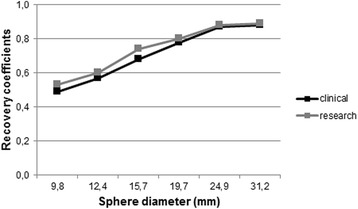
Recovery coefficients for the six spheres in the small Jaszczak phantom with a sphere-to-background ratio of 4.4:1

For both reconstruction methods (“clinical” and “research”), all spheres in the Jaszczak phantom could be visually discerned (Fig. 3). The image does show a small elliptical distortion of the spheres, consistent with the spatial dependency of the spatial resolution. Recovery coefficients ranged from 0.49 to 0.88 for the clinical reconstruction and from 0.53 to 0.89 for the research reconstruction with increasing CRC for increasing diameter (Fig. 4).

### Visual assessment of spatial resolution

Upon visual inspection of the reconstructed slices showing cold rods of the Jaszczak phantom, the smallest rod group that could be discerned was the 6-mm rod group (Fig. 3), for both reconstruction methods.

### Uniformity

**Fig. 5:**
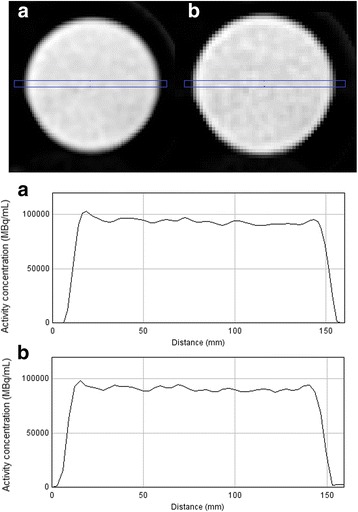
Top: reconstructed images (sum of four slices) of the uniform compartment of the Jaszczak phantom, for the clinical reconstruction (**a**) and the research reconstruction (**b**) algorithm. Bottom: activity concentrations profile across the summed slices as indicated in the displayed images (in blue)

Visually, reconstructed images are uniform and profiles are horizontal and show limited variability (Fig. 5). CV was 2.6% for the clinical reconstruction and 3.0% for the research reconstruction.

### Striatal phantom

**Fig. 6:**
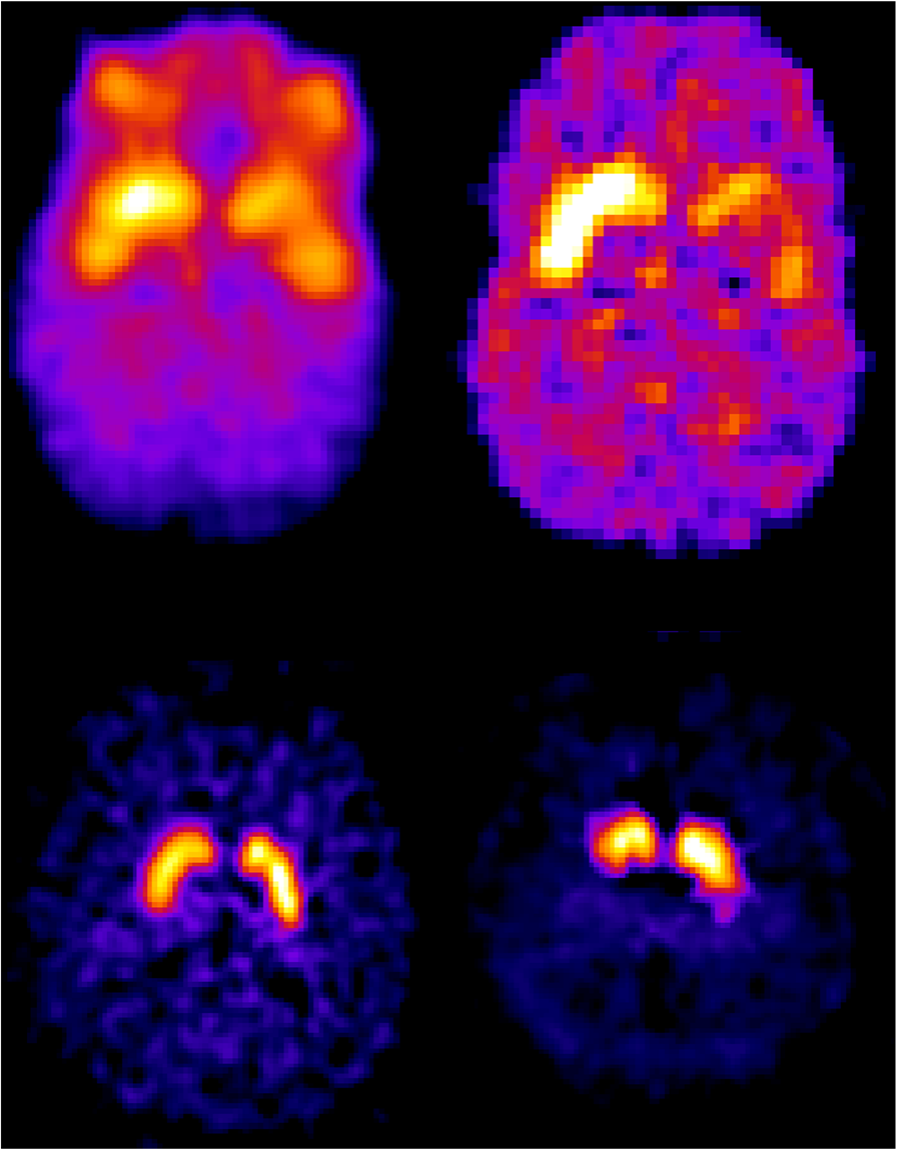
Top: striatal phantom DAT images with a right striatal-to-background ratio of 4.6:1 and a left striatal-to-background of 3.5:1. Left image shows the clinical reconstruction, and right the research reconstruction. Below: a typical example of a [^123^I]ioflupane SPECT scan acquired 4 h post-injection from a healthy subject (left) and a patient with striatal dopaminergic deficit due to Parkinson’s disease (right). Subjects were injected with ~ 111 MBq [^123^I]ioflupane. Only the clinical reconstruction is shown

Figure 6 shows the reconstructed images of the striatal phantom with the striatal-to-background ratio of 4.6:1 and 3.5:1. For the clinical reconstruction algorithm recovery coefficients were 0.55 for the right and 0.51 for the left striatum. For the research reconstruction, these were 0.54 and 0.50 for right and left striatum. Linearity of quantitative values was observed; represented by a Pearson’s *R* of 0.97 (Additional file 3: Figure S3).

### Typical example clinical image

Symmetric and intense ^123^I-ioflupane binding in the caudate nucleus and putamen was observed in the healthy control. In the patient with a clinical diagnosis of PD, the binding was asymmetrical and lower in the putamen than in the caudate nucleus, as expected [2] (Fig. 6).

## Discussion

The present results demonstrated acceptable image quality for the InSPira HD. The acquired SPECT images showed detailed information with good resolution, contrast, and uniformity. Small variations in spatial resolution across the FOV were observed, likely due to the geometry of the system. Recovery coefficients were lower than expected, but linearity of quantitative results was observed.

### NEMA standard testing

The aim of the present study was to reproduce previous findings [6] and to evaluate additional phantom measurements relevant for DAT imaging. Therefore, in addition to typical NEMA testing setup for gamma cameras (NU 1-2012), several tests were adapted from NU 2-2012, originally designed to evaluate performance of PET systems, to augment conventional SPECT testing. Measurements on recovery contrast and reconstructed uniformity (all NU 2-2012) are highly relevant for high-resolution brain imaging. Importantly, due to the unique hardware configuration of the InSPira HD system, several adjustments had to be made to standard testing. The phantom tests included in the paper are therefore not a representation of a generic performance evaluation of a novel SPECT system. First, due to the small bore size, a smaller, custom made, Jaszczak phantom had to be used. Second, standard NU 1-2012 includes planar imaging (planar field uniformity and planar spatial resolution) in addition to SPECT imaging, which is not possible for the InSPira HD. Third, standard NEMA testing often includes filtered back projection as the recommended reconstruction approach. Since this method is not available for the InSPira HD, all reconstructions were performed with the proprietary iterative reconstruction algorithms that were supplied by the manufacturer, which includes the algorithm that will be used clinically. Other variables such as matrix size, scatter correction, and scan time all deviated from standard testing. Finally, sensitivity measures were not performed. Sensitivity is an important parameter for system performance since it provides information on acquisition time and associated noise, with respect to achieved spatial resolution (Madsen et al. [10]; Jansen and Van der Heyden [11]). Due to the geometry of the InSPira HD, a unique metric should have been developed specific for the system, making interpretation of the results difficult. It was therefore decided not to include sensitivity measures in the present manuscript. The results of the present manuscript do show that sufficient contrast and signal to noise ratios are achieved at clinically relevant tracer doses and acquisition time, which is an indication that sensitivity is not a problem for this system. Overall, the results described in this paper give an important impression on performance of the InSPira HD system in light of DAT imaging. However, since major adjustments had to be made to standard NEMA testing setup, direct comparison to other SPECT systems is not desirable.

### Image quality

Using the high-resolution reconstruction approach resolution around 3 mm FWHM was reached for phantom sources in air. In comparison, conventional SPECT systems report spatial resolution around 10 mm [12]. This high-resolution reconstruction showed stable resolution across the FOV, in contrast to the other two reconstruction methods (see below for further discussion on this issue). Due to the high number of iterations, this approach was included in the present analyses solely to test the limits of the system. With 1000 iterations, reconstruction times are too long (up to ~ 15 h) for clinical data, and image noise is likely to increase with increasing number of iterations [13]. Two other reconstruction approaches (research and clinical reconstruction) were examined. Both approaches yielded very similar results. Evaluation of reconstructed slices (with attenuation correction) from the uniformity section of the Jaszczak phantom showed good uniformity with CV values < 3%, indicating low noise and accurate attenuation correction. Spatial resolution in air was good with resolution of approximately 5–6 mm near the center of the FOV and 9–11 mm at the edge of the FOV. Furthermore, upon inspection of the rods section of the reconstructed Jaszczak phantom scan, the ≥ 6-mm rods could be visually resolved. Recovery coefficients indicated good contrast for the Jaszczak phantom for both reconstruction approaches; CRC ranging from 0.49 to 0.89, with increasing CRC for increasing diameter. Overall, the research reconstruction seemed to perform slightly better with respect to resolution and contrast, whereas the clinical reconstruction scored slightly better with respect to uniformity.

### Image quality issues

Despite overall good image quality, some issues were identified. Spatial resolution in *x* and *y* direction in the center of the FOV, and on left and right sides seemed to differ slightly. Overall, these differences were not significant and likely representative of standard measurement errors; phantoms were placed within the center of the FOV using a laser line, but perfect placement proved to be difficult. Results from spatial resolution in water did differ from measurements in air. This is not uncommon for iterative expectation maximization algorithms; convergence is non-linear and the reconstructed spatial resolution depends on several parameters such as local contrast and image count density [13]. The unique geometry of the InSPira HD camera is designed to provide high resolution across the FOV, however, the measurements revealed changing spatial resolution as a function of distance to the center of the FOV. In reconstructed images, a radial elongation artifact or tear-shape effect was observed for circular objects, likely a result from the changing spatial resolution across the FOV. Variation in spatial resolution across the FOV is not uncommon for systems with such geometry. The radial elongation effect was also observed in the measurements using line sources in water including background concentrations: significant differences in spatial resolution *x* and *y* direction were observed for the two sources outside the FOV. Although an important limitation of the InSPira HD system, for clinical DAT imaging this is not necessarily a problem. Since when imaging a patient (if positioned correctly), the striatum will be located close to the center of the FOV, resolution for this region will be optimal and image distortions should be minimal. In the analysis of DAT images, the occipital cortex or cerebellum is used as reference region, which will be located somewhat further away from the center of the FOV. Therefore, some blurring and distortion of this region is to be expected. For its use as reference region, where a global average is calculated, we believe this will not be a problem.

Additionally, striatal uptake ratios were lower than expected, with recovery coefficients around 0.5. Importantly, linearity of quantitative results was observed. Therefore, in order to assess if patients’ ratios deviating from ‘normal,’ an age-matched control database should be used, that should be cross-calibrated with an independent sample of healthy subjects scanned on the InSPira to account for relatively low striatal uptake ratios. Hereby, adhering to the guidelines published by the European Association of Nuclear Medicine and Society of Nuclear Medicine and Molecular Imaging guidelines [9].

Finally, for the majority of our phantom studies, the phantoms were filled with ^99m^Tc and not with ^123^I, although we primarily focused on the suitability of the InSPira HD system for DAT SPECT imaging with ^123^I-ioflupane. However, this system will also be used for ^99m^Tc-labeled radiotracers like ^99m^Tc-HMPAO. Nevertheless, one has to take into account that the image quality for ^99m^Tc-labeled tracers may be better than for ^123^I-labeled tracers due to the different physical properties of both radionuclides, but fortunately the ^123^I-ioflupane scans obtained in human subjects and the ^123^I-filled striatal phantom experiments showed a good image quality.

## Conclusions

Although some image quality issues were identified, performance measurements demonstrated acceptable image quality of the InSPira HD for DAT SPECT imaging in humans.

## Additional files

Additional file 1: Set-up of phantom experiments. A: Point sources in air in the coronal plane (dotted lines represent x- and z-axis). B: Line sources in air in transverse plane (dotted lines represent the round acrylic disk from which the line sources point out). C: Point sources in transverse plane (inserted in a cylinder filled with water). D: Jaszczak phantom with spheres in transverse plane. E: Jaszczak phantom with rods insert in transverse plane. (TIFF 219 kb)

Additional file 2: Representative SPECT images of the phantom measurements. Point sources in air (A), point sources in water with no background concentration (B), point sources in water with 0.1% background concentration (C), and point sources in water with 1% background concentration (D). The reconstruction approaches are shown as following: clinical reconstruction on the left, research reconstruction in the middle, and the high resolution reconstruction on the right (only available for A).(DOC 117 kb)

Additional file 3: Figure S3. Scatterplot of true striatal ratio's (x-axis) and measured striatal ratio's (y-axis), for the striatal phantom measurements. (TIF 10 kb)

## Abbreviations

^123^I-FP-CIT: ^123^I-Ioflupane
ACR: American College of Radiology
AMC: Academic Medical Center
BRASS: Brain Registration and Analysis Software Suite
CRC: Contrast recovery coefficients
CT: Computed tomography
CV: Coefficient of variation
DAT: Dopamine transporter
DLB: Dementia with Lewy bodies
FOV: Field of view
FWHM: Full width at half maximum
MAP: Maximum a posteriori
MSA: Multiple-system atrophy
NEMA: National Electrical Manufacturers Association
PD: Parkinson’s disease
PSF: Point spread function
PSP: Progressive Supranuclear palsy
ROI: Region of interest
SPECT: Single-photon emission computed tomography
VOI: Volume of interest
