# Supplementary figures and images for: Performance evaluation of a novel brain-dedicated SPECT system
Source: EJNMMI Phys. 2018 Mar 1;5:4. doi: 10.1186/s40658-018-0203-1 (PMC5833889; doi:10.1186/s40658-018-0203-1)

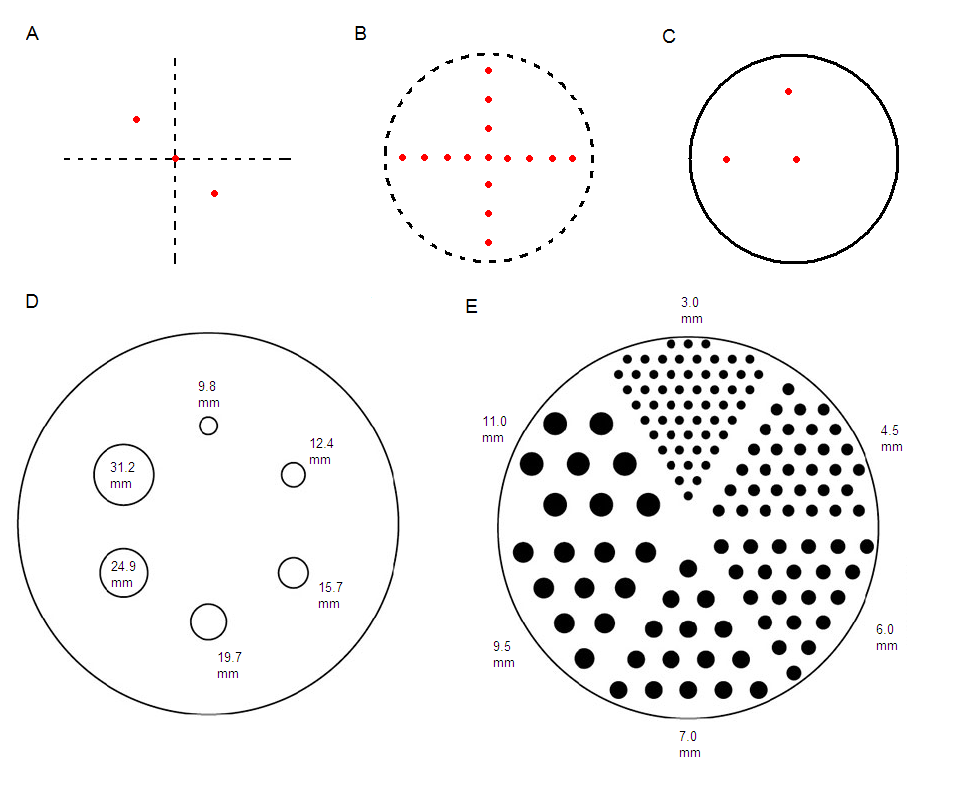

Supplement: Supplementary file 1 — Set-up of phantom experiments. A: Point sources in air in the coronal plane (dotted lines represent x- and z-axis). B: Line sources in air in transverse plane (dotted lines represent the round acrylic disk from which the line sources point out). C: Point sources in transverse plane (inserted in a cylinder filled with water). D: Jaszczak phantom with spheres in transverse plane. E: Jaszczak phantom with rods insert in transverse plane. (TIFF 219 kb) [file 40658_2018_203_MOESM1_ESM.tif]

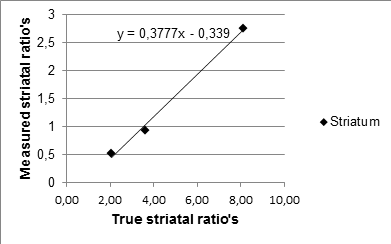

Supplement: Supplementary file 3 — Scatterplot of true striatal ratio's (x-axis) and measured striatal ratio's (y-axis), for the striatal phantom measurements. (TIF 10 kb) [file 40658_2018_203_MOESM3_ESM.tif]
