# Supplementary material for: Performance evaluation of a novel brain-dedicated SPECT system
Source: EJNMMI Phys. 2018 Mar 1;5:4. doi: 10.1186/s40658-018-0203-1 (PMC5833889; doi:10.1186/s40658-018-0203-1)

Figure S2; Representative SPECT images of the phantom measurements. Point sources in air (A), point sources in water with no background concentration (B), point sources in water with 0.1% background concentration (C), and point sources in water with 1% background concentration (D). The reconstruction approaches are shown as following: clinical reconstruction on the left, research reconstruction in the middle, and the high resolution reconstruction on the right (only available for A).


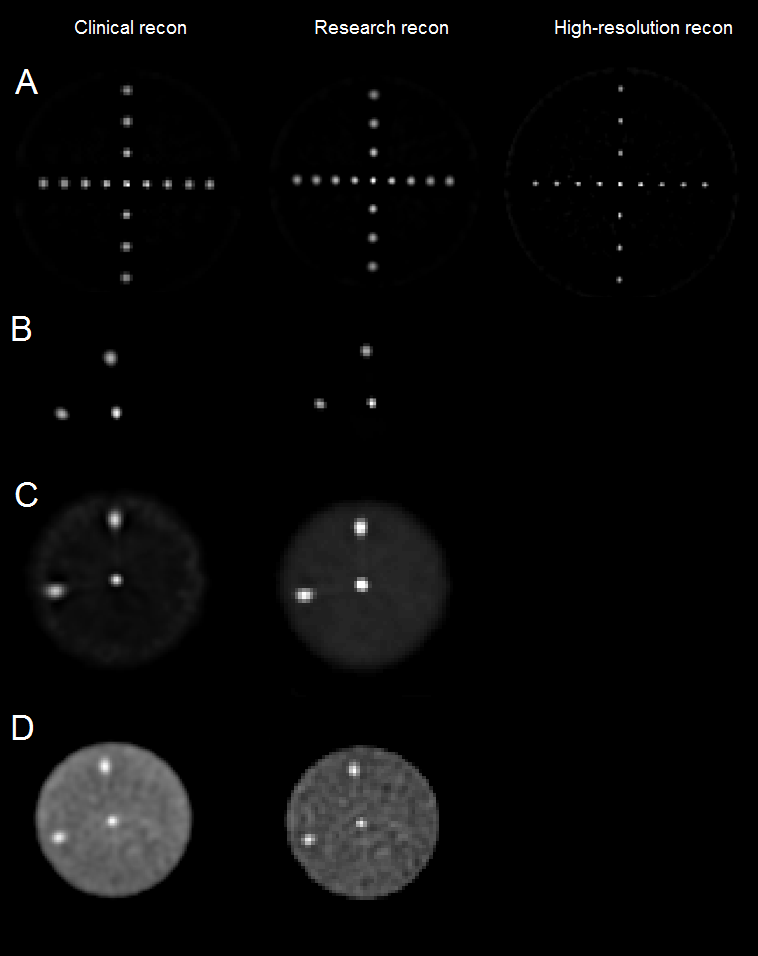

Supplement: Supplementary file 2 — Representative SPECT images of the phantom measurements. Point sources in air (A), point sources in water with no background concentration (B), point sources in water with 0.1% background concentration (C), and point sources in water with 1% background concentration (D). The reconstruction approaches are shown as following: clinical reconstruction on the left, research reconstruction in the middle, and the high resolution reconstruction on the right (only available for A).(DOC 117 kb) [file 40658_2018_203_MOESM2_ESM.doc]
